# New Fabrication Method of Silicon Sub-Micron Beams with Monolithic Contacts for Thermoelectric Transport Properties Analysis

**DOI:** 10.3390/nano12081326

**Published:** 2022-04-12

**Authors:** Andrej Stranz, Marc Salleras, Luis Fonseca

**Affiliations:** 1 Pronawo UG, Allee der Kosmonauten 26, 12681 Berlin, Germany; 2 Institute of Microelectronics of Barcelona, IMB-CNM (CSIC), C/Til·lers s/n—Campus UAB, Bellaterra, 08193 Barcelona, Spain; marc.salleras@imb-cnm.csic.es (M.S.); luis.fonseca@imb-cnm.csic.es (L.F.)

**Keywords:** silicon, thermoelectric, microdevice, CMOS fabrication

## Abstract

Micromachined devices were developed and fabricated using complementary metal-oxide-semiconductor (CMOS)/micro-electro-mechanical systems (MEMS) technology allowing for the analysis of transport properties of silicon sub-micron beams having monolithic contacts. The beams were fabricated by a combination of deep reactive ion etching (RIE) and potassium hydroxide (KOH) etching techniques on standard p and n silicon bulk and silicon-on-insulator (SOI) wafers. Simultaneous fabrication of many devices on one wafer allows for the extraction of statistical information to properly compare the different layers and contacts. Fabricated devices are presented, underlining the feasibility of the proposed microdevice. The methods used to manipulate the geometry and the surface roughness of the single crystalline silicon beams are described. The presented measurement device offers the possibility to determine simultaneously all the main transport values, thermal, and electrical conductivities as well as the Seebeck coefficient.

## 1. Introduction

In applications, such as the Internet of Things (IoT)-networks, wearable devices, and lab-on-chip modules, there is a need for electrical power of less than 1 mW to drive the autonomous units or to detect heat from chemical reactions for medical applications [1,2,3]. One possible, sustainable way to provide this energy is to harvest waste energy from the environment. There are several possibilities to harvest the available energy and convert it into electrical energy, e.g., from vibrations, radiation, and thermal gradients. In this paper, sub-micron single crystalline silicon beams fabricated from commercially available p- and n-type wafers are presented. Such beams can be implemented in thermoelectric microgenerators to convert thermal energy from the environment into electricity, providing power for autonomous devices. Especially in those situations where no vibrations or solar radiation are present, energy conversion from thermal gradients might be the only feasible option to provide electrical power to devices.

Silicon-based nanostructures have been studied intensively in research as an alternative to materials currently used in thermoelectric applications [4,5,6,7]. From the investigations described in [5], the power density of the silicon NW-based microgenerator was obtained to be 41.2 µW cm^−2^. Silicon is compatible with state-of-the-art electronics manufactured in large volumes, i.e., technologies for processing are available offering a well-developed infrastructure. Additional advantages of silicon, such as its high availability as raw material, no health hazards, and high environmental compatibility, justify further research and development efforts for its implementation in thermoelectric applications.

Despite its many advantages, the main challenge of silicon as a material for thermoelectric applications is to reduce the high thermal conductivity of single crystalline silicon, while keeping a high electrical conductivity and high Seebeck coefficient. A possible strategy is to decrease its intrinsic thermal conductivity by reducing the geometry in one or two dimensions. A further decrease is achieved by the introduction of surface roughness or micropores in the nanometer range [8,9,10,11,12]. In bulk single crystalline silicon, the thermoelectric transport properties of electrical conductivity, thermal conductivity, and Seebeck coefficient depend on the doping level and temperature [13,14,15,16]. The figure of merit *ZT* is used to evaluate the performance of the material for thermoelectric applications.

$$ZT=\frac{S^{2}\sigma }{\kappa }T$$

where *S* is the Seebeck coefficient, *σ* is the electrical conductivity, and *κ* is the thermal conductivity. It has been reported in the literature that heavily doped bulk silicon shows an order of magnitude higher *ZT* values at room temperature compared to lightly doped silicon [17,18]. The improvement is caused by higher electrical conductivity *σ* and thus higher power factor *S*^2^*σ* for heavily doped silicon, and reduced thermal conductivity due to enhanced phonon-impurity scattering. An additional reduction in the thermal conductivity can be achieved by tailoring the geometry and surface of single-crystalline bulk silicon beams and nanowires, which further improves the thermoelectric performance of this material. As the energy to be harvested is waste heat at no cost, a moderate reduction in thermal conductivity might be enough to justify the use of single-crystal silicon in thermoelectric harvesting applications. Other factors like material availability, processing costs, and its extremely mature fabrication technology outweigh a less optimal *ZT* value for silicon. The importance of fabrication flexibility is based on the fact that, for micro energy harvesting applications, expensive heat exchangers should be avoided. Therefore, device parameters, such as the active area or the length of the thermoelectric (TE)-legs, should be easily adjustable to fit the existing thermal conditions in the given application. 

The transport properties in crystalline silicon can be significantly changed by nanostructuring. Most approaches focus on the reduction in thermal conductivity by increasing phonon scattering at interfaces and surfaces. The phonon mean free path in single crystalline silicon is about 250 nm at room temperature [19]. In silicon nanostructures, such as nanowires with a diameter below 250 nm, the propagation of phonons at room temperature is significantly disturbed by scattering at the nanowire surface. This results in reduced thermal conductivity in such one-dimensional structures. On the other hand, the electrical conductivity is not affected, since the mean free path of electrons is only about a few nm at room temperature [20]. Accordingly, the reduction in thermal conductivity in silicon nanowires is experimentally confirmed and theoretically supported by several groups [21,22,23,24]. The ZT value for silicon nanowires is determined to be 0.7 at room temperature, shifting silicon into the range of bismuth telluride, the state-of-the-art thermoelectric material. Thus far, experimental evidence relies on the analysis of single nanowires fabricated using vapor–liquid–solid (VLS) processes, electroless etching (EE), or electron beam lithography (EBL) followed by reactive ion etching (RIE). An additional way to reduce the thermal conductivity, which is discussed in the literature, is to increase the phonon scattering by roughening the sidewalls or surfaces of nanostructures [25,26,27].

One of the most applied methods to measure the transport properties of nanostructures is to place them between two suspended membranes [28,29,30,31,32,33]. Using focused ion beam (FIB)-technology, the nanostructures, mostly nanowires or nanoribbons, are contacted with the metal on the suspended platforms by deposition of platinum. The majority of the reports found in the literature describe an approach where the measuring microdevice and the nanostructures to be measured are fabricated separately and combined subsequently into a measurement system. This report presents a fabrication method of microdevices with integrated silicon beams for transport properties analysis. Furthermore, the proposed microdevice can be deployed to analyze the transport properties of materials like nitrides, oxides, or metals which are deposited using CMOS-compatible processes on silicon wafers. By following the proposed time-controlled fabrication scheme, the material of interest can be completely suspended and characterized while keeping monolithic contacts between bulk and nanostructure.

## 2. Materials and Methods

### 2.1. Materials

**Figure 1:**
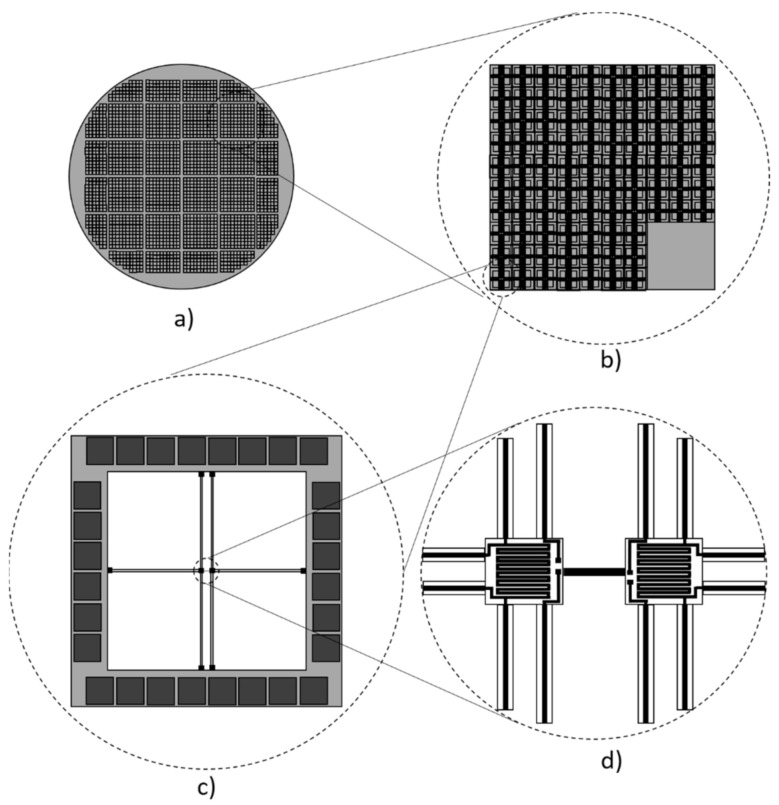
Schematic of the layout concept of microdevices with suspended platforms for manufacturing out of one 4″ bulk- and SOI wafer. (**a**) Arrangement of units on the wafer. (**b**) Regular unit with microdevices. (**c**) Single microdevice. (**d**) Two platforms with a silicon beam in between and supporting bridges.

Off-the-shelf <100> oriented, 4-inch bulk silicon and SOI wafers (Ultrasil Corporation, Hayward, CA, USA) were used for the fabrication of silicon beams embedded between two platforms to investigate the impact of the geometry, contacts, and surface roughness of those beams. The boron- and phosphorus-doped, double-side polished bulk silicon wafers with a nominal resistivity for n-type wafers of 145–290 Ω·cm and for p-type wafers of 4–400 Ω·cm had a thickness of (300 ± 10) µm. Both SOI wafers had a device layer of (5 ± 0.5) µm and a resistivity of 7–13 Ω·cm for p-type and 1–10 Ω·cm for n-type layer, respectively. The boron-doped p-type SOI wafer had a BOX layer thickness of (1 ± 5%) µm and the phosphorus-doped n-type SOI wafer had a BOX layer thickness of (2 ± 5%) µm. The handle layer of the p-type SOI wafer had a thickness of (523 ± 25) µm and the n-type wafer of (510 ± 25) µm. The realization of the devices has benefited from the well-developed and established fabrication line in a CMOS/MEMS cleanroom, where all manufacturing steps were well orchestrated. The available space on a 4-inch wafer allowed the fabrication of more than 1500 microdevices on a single wafer. This large number of devices allowed us to identify and analyze measurement errors that might occur due to, e.g., material inhomogeneity, fabrication uncertainties, or metal-silicon contact deviations. Thinking towards the realization of silicon-based thermoelectric modules, n- and p-type doped semiconductors are required. High commercial availability of n- and p-type bulk and SOI silicon wafers with different doping levels enable to meet this requirement, especially cost-effective bulk silicon, which might be of interest for industrial applications. Figure 1 shows schematically the structures fabricated from a 4-inch wafer.

### 2.2. Device Fabrication

On a single 4-inch wafer, 32 test units were realized (Figure 1a). In the main center area of the wafer, 16 regular units were arranged. An additional 16 units containing fewer microdevices were arranged on the sides of the wafer to optimize the utilization of the wafer area. Each regular unit consists of 91 microdevices and a device-free area for handle purposes (Figure 1b). One single microdevice has dimensions of (1.5 × 1.5) mm^2^. Aside from the regular devices described below, devices with shorter supporting bridges, as well as structures to analyze the metal-semiconductor contacts and electrical conductivity, are fabricated. For measurements or further processing steps, single units can be separated from the wafer if required. The silicon beams between the suspended platforms in the center of the device are designed to have a width of 8 µm. The length of the beams varies within the unit. There are beams with lengths of 20 µm, 30 µm, and 40 µm. The variation of the beam length can be used to investigate the linearity of the transport properties as a function of the surface texture since the scattering mechanisms inside the material shows other characteristics compared to scattering on surfaces. Even the scattering on different types of rough surfaces differs [34,35].

The microdevice is built up of a frame on which twenty-eight contact pads are located with dimensions of (150 × 150) μm^2^ each (Figure 1c). The pads can be contacted with probes to apply electrical current and measure the voltage drop. Additionally, four extra microdevices are distributed uniformly within the unit where the suspended platforms are connected by beams having the same width as the platforms (42 µm). These beams are expected to have bulk properties due to their dimensions and can be used as a reference structure to determine the thermal contact resistance between the platinum (Pt) heater, deposited silicon nitride (SiN) layer, and silicon platform. Furthermore, another four microdevices are distributed uniformly within the unit without any beams between the platforms. Those devices serve as a reference to determine the heat flux through the silicon nitride/platinum (SiN/Pt) supporting bridges that connect the platforms to the frame. The fabrication of identical devices distributed all over the wafer provides statistical analysis of the data specifying measurement errors.

As shown in Figure 1c, each of the two suspended platforms in the center is connected with six SiN/Pt supporting bridges. Four platinum leads are intended for the measurement and subsequent calculation of the platform’s temperature in a four-wire configuration by using the temperature coefficient of resistance of the deposited platinum sensors. Two leads are used to contact the silicon beneath the SiN isolating layer. Together with the two connections on the opposite platform, the I–V curves of the silicon beams between the platforms, as well as the Seebeck voltage, can be determined. Both platinum serpentine coils on the platforms can be used, both as sensors and as heaters. Temperature sensors are also located at the four anchor regions on the frame, where the SiN/Pt supporting bridges connect the platforms to the frame, to verify the frame temperature. The platinum resistance coils on the platforms and the frame have an overall length of 300 μm, a width of 2 µm, and a thickness of 0.3 µm.

The platforms are (38 × 42) μm^2^ in size and the 6 SiN/Pt supporting bridges are (475 × 6 × 0.3) μm^3^.

**Figure 2:**
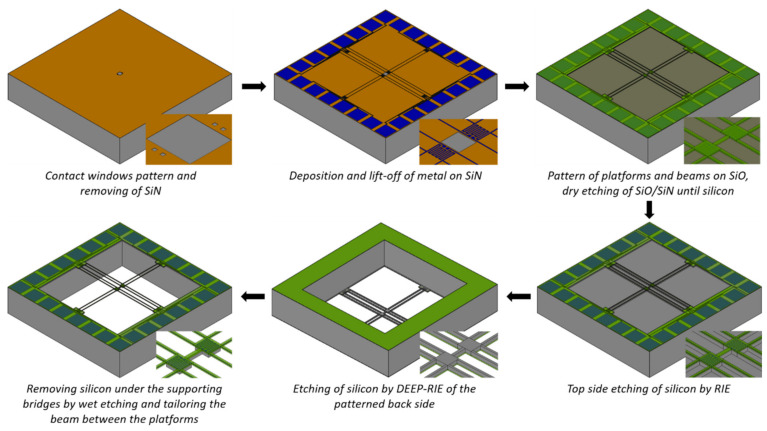
Schematic of a fabrication sequence for the microdevice with suspended platforms and beams.

The fabrication sequence is depicted in Figure 2. In the beginning, a 0.3 µm layer of SiN is deposited on the silicon wafer by low-pressure chemical vapor deposition (LPCVD). The contact windows and the area between the platforms are patterned using optical lithography, and the SiN is removed by dry etching. Subsequently, the photoresist is spun on the wafer, and patterned for the chromium/platinum (Cr/Pt) metallization (30/300 nm) using a lift-off process. After the lift-off is completed, 300 nm of silicon oxide (SiO) is deposited by LPCVD on top of the wafer. The resulting structure on the top side of the wafer is used for the pattern alignment on the back-side of the wafer by double-side photolithography, where large openings of the mask material for the silicon back-side etching will be eventually defined. The subsequent photoresist patterning on the top side defines the platforms, the silicon beam between the platforms, and the supporting bridges’ geometries. After that, dry etching of SiO/SiN is performed until the silicon is reached. The exposed silicon is further etched by dry etching for a given time.

**Figure 3:**
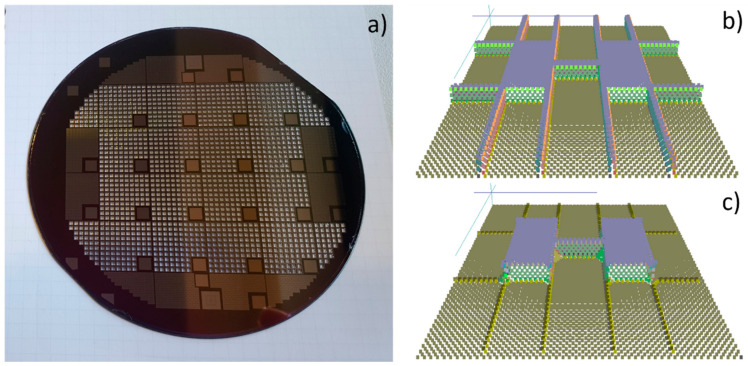
(**a**) SOI wafer after the final back-side dry etching to suspend the platforms. (**b**) Wet etching simulation of silicon in KOH solution for the proposed microdevices after the final dry etching. Initial geometry before wet etching. (**c**) Simulated results for the geometry after 20 min. in KOH-based etchant.

In this step, the height of the beams is defined according to the duration of the etching procedure for bulk silicon samples. On the other hand, for SOI samples, the thickness of the wafer device layer defines the height of the beams since the etching process stops at the buried oxide (BOX) layer. In the next step, the dry etching of the wafer from the backside is performed. After the back-side etching of silicon, the platforms are suspended, and they are supported by six Si/SiN/Pt/SiO arms in the case of bulk silicon samples. In the case of SOI samples, the BOX layer is removed after the back-side silicon etching. In this step, the SiO layer on the top is also removed. For the bulk silicon samples, the SiO layer on the top is removed by hydrofluoric acid (HF) as well, before KOH etching. Hereafter the silicon is still present under SiN/Pt supporting bridges. To finally suspend the SiN/Pt arms, the silicon below is removed by wet chemical etching using a KOH solution. For allowing the entire under-etching of the supporting bridges and simultaneously, the reduction in the width of the silicon beam between the platforms, the designs were rotated by 45 degrees in the (100) oriented wafers (Figure 3 left). After dry etching, the rotated design resulted in exposing the (110) oriented silicon sidewalls under the supporting bridges, as well as the sidewalls of the beams between the platforms. Exposed to KOH solution, the (110) oriented silicon crystal plane is etched perpendicular to the surface of the sidewalls, removing silicon under the supporting bridges from both sides (Figure 3 right). While the silicon under the supporting bridges is completely removed, the width-reduced silicon beam between the platform remains after KOH etching. This is ensured by designing the starting width of the beams between the platform to be 2 µm wider than the silicon under the SiN/Pt supporting bridges. 

After removing the silicon under the supporting SiN/Pt bridges, the remaining silicon beam between the platforms can be exposed to KOH solutions of different concentrations and at different temperatures to tailor its roughness.

Experiments show that the surface roughness of silicon after KOH etching depends on the concentration and the solution temperature [36]. Careful application of the time-dependent reduction in the silicon beam width and surface texture engineering opens an opportunity to analyze the modification of transport properties when going from bulk to nano, or the effect of nanofeatures on bulk structures.

## 3. Results and Discussion

**Figure 4:**
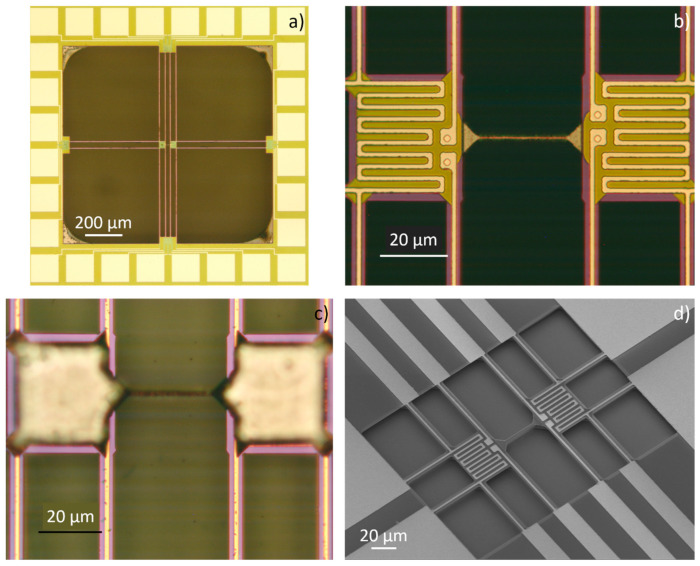
(**a**) General top view of a suspended device manufactured from an SOI wafer. (**b**) Platforms with a silicon beam between them, after removing the silicon under the supporting SiN/Pt bridges with KOH. (**c**) Back-side view of the platforms indicating successful removal of silicon. (**d**) General top view of a “small” device with shorter supporting beams after BOX layer and KOH etching (no stiction was observed).

Figure 4 shows SOI devices after suspending the platforms and removing the silicon by KOH solution under the SiN/Pt supporting bridges. The BOX layer, as well as the protective oxide layer on top, are etched by buffered HF. Figure 4a shows the top view of the device with structured metallization and contact pads. A closer view of the two platforms with the silicon beam in between is shown in Figure 4b. Here, the serpentine structure of the platinum lines for heating and sensing, as well as the four-wire arrangement to measure the voltage drop over the serpentine structures, are clarified. In Figure 4b, the four contacts to the silicon under the silicon nitride layer are also visible. Their purpose is to measure the electrical potential. The resulting voltage drop across the silicon beam as a result of a temperature difference between the platforms (Seebeck voltage) can be measured. Additionally, the electrical resistance of the beam can be calculated by applying a current through the silicon beam and measuring the corresponding voltage drop. Figure 4c shows the platforms from the back-side. The silicon under the SiN/Pt supporting bridges is removed and the Pt lines are observable through the silicon nitride. Since the etching rate of KOH depends on the type and doping level of silicon, it was not possible to use a fixed time for all samples. To verify the progress of silicon removal under the supporting bridges, the samples were checked by removing them from the etchant and investigating them under the microscope at different time intervals. The removal of silicon under the supporting bridges is a time-dependent process. The etching rate of <110> silicon is expected to be approx. 100 µm/h [37] for 40% KOH concentration at 80 °C.

For better control of the etching process, the temperature was reduced to 40 °C. This decreased the etching rate nominally to 10 µm/h. As already mentioned, the silicon beams between the platforms were designed to be 2 µm wider compared to the supporting bridges. After the complete removal of the silicon under the latter, silicon beams between the platforms were found to have widths from 1.1 µm to 1.8 µm. This is smaller than the expected value of 2 µm. The reason for this is the different doping types and levels of wafers. The doping type and level of silicon affect the etching rate. Figure 4d shows a SEM image of an SOI test device with short SiN/Pt supporting bridges. For these devices, the back-side was not dry-etched to suspend the platforms. The platforms were suspended by etching the BOX layer. As in the case of bulk silicon, the removal of the silicon under the supporting SiN/Pt bridges and the width reduction in the silicon beam between the platforms is done by a time-controlled etch in the KOH solution. Such devices, with shorter supporting beams, are more mechanically stable and were used for additional tests. 

**Figure 5:**
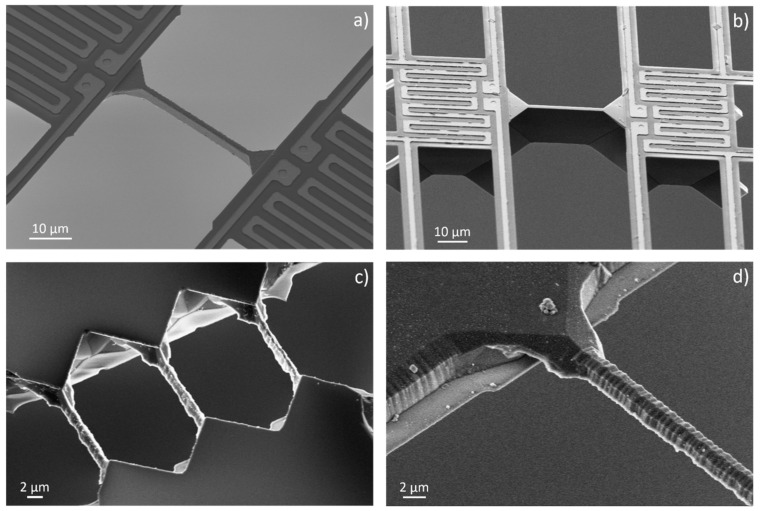
Micro platforms with silicon beams in between are fabricated from single-crystalline bulk (**a**,**b**) and from SOI (**c**,**d**) silicon wafers. (**a**) Top view of an n-type silicon device with a height of 4 µm. (**b**) Top view of an n-type silicon device with a height of 17 µm. (**c**) Back-side view of a p-type silicon device with three silicon beams in parallel connecting the platforms. (**d**) Back-side view of a p-type silicon beam that is shaped into a rod after wet etch roughening.

Figure 5a,b show n-type silicon beams of different lengths and heights after silicon removal under the supporting beams. These microdevices were fabricated from bulk silicon wafers. In contrast to devices fabricated out of SOI wafers, where the height of the beams is defined by the device layer thickness, the height of the beams made of bulk silicon wafers is adjusted by the duration of the dry etching step.

The etching duration from the top side, as well as from the bottom side, defines the beam height. After the KOH wet etching, smooth sidewalls of the silicon beams were found, as was expected from etching with 40% concentrated KOH solution. To further reduce the beam widths or increase the surface roughness, lower concentrations of KOH solutions were used. The solution concentration was changed to 20% while keeping the temperature at 40 °C. The results of the etched beam from n-type and p-type SOI samples are shown in Figure 5c,d, respectively. As the etching process progressed, the silicon beams were shaped into a rod. The surface of the rod was much rougher compared to the smooth surface of the beams, which were etched with a solution of 40% KOH.

**Figure 6:**
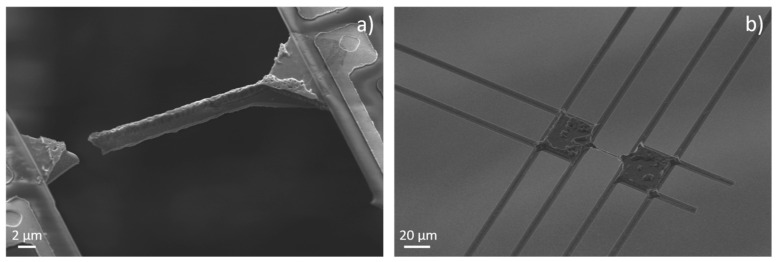
(**a**) Broken silicon beam between the platforms due to the internal tensile stress. (**b**) A device with two broken supporting SiN/Pt bridges reducing the internal tensile stress inside the silicon beam and enabling further reduction in the cross-sectional area of the silicon beam.

The continuous reduction in the beam cross-sectional area by KOH solution resulted in higher tensile stress in the silicon beams. The tensile stress is caused by the SiN/Pt supporting bridges oriented in the direction of the silicon beams. The tensile stress inside the supporting bridges induced during the thermal deposition of the material tears apart the silicon beams between the platforms when the cross-section area is reduced. The critical width of the silicon beams to withstand the tensile stress was observed to be about 1 µm. Figure 6a shows an example of a broken beam after etching with a lower concentrated KOH solution. An example of a device where a couple of supporting bridges oriented in the beam direction between one platform and the frame were broken is presented in Figure 6b. In this device, it was possible to reduce the diameter of the silicon beam between the platforms below 1 µm, avoiding the beam breaking.

From this observation, it can be concluded that the platforms should be designed to be anchored to the frame on three sides in a T-like arrangement, or even only on two sides (supporting bridges perpendicular to the silicon beam) to reduce the internal tensile stress inside the silicon beams for the fabrication of thinner structures.

## 4. Conclusions

In this report, a new cost-efficient method to fabricate single crystalline silicon beams embedded in a characterization device for the analysis of thermoelectric transport properties is introduced. The concept and the fabrication flow are described in detail. The approach of monolithic contacts between bulk and sub-micron silicon beams enables the realization of the minimum possible contact resistance. The method offers an opportunity to analyze the transport properties of silicon structures reducing the dimensions continuously from micro to nano. The introduced microdevice allows for the investigation of the transport properties in both directions by switching the function of the platforms between the heater and temperature sensor. Furthermore, the impact of the surface texture on transport properties can be investigated through the adjustment of the surface roughness using a simple method based on the concentration variation of the KOH solution. It was shown that not only SOI wafers but also 4-inch off-the-shelf bulk wafers can be used to manufacture suspended devices. This fact, and the avoidance of time-consuming e-beam lithography and FIB techniques, make this fabrication method more cost-effective. The internal tensile stress of the SiN/Pt supporting bridges was identified as the root of the limitation for the minimum silicon beam diameter achievable, slightly below one micrometer, before breaking. To avoid the beam breaking due to the internal stress of the SiN/Pt supporting bridges and to enable further reduction in the beam width towards a few hundred nm or even below, it is necessary to redesign the device in a way that the tensile load in the beam is avoided. 

## Data Availability

All relevant data are provided in the article.
